# US workers' willingness to accept meatpacking jobs amid the COVID‐19 pandemic

**DOI:** 10.1002/jaa2.8

**Published:** 2022-04-27

**Authors:** Jeff Luckstead, Rodolfo M. Nayga, Heather Snell

**Affiliations:** ^1^ School of Economic Sciences Washington State University Pullman Washington USA; ^2^ Department of Agricultural Economics Texas A&M University College Station Texas USA; ^3^ Programming Specialist Arvest Bank Bentonville Arkansas USA

**Keywords:** COVID‐19, discrete choice model, job attributes, meatpacking employment

## Abstract

We implement a discrete choice experiment to examine the impact of COVID‐19 exposure risk, unemployment risk, enhanced and extended unemployment benefits, and job attributes on low‐skilled workers' willingness to accept (WTA) meatpacking jobs. With a sample average WTA wage of $22.77/h, the current national average meatpacking wage of approximately $15/h is too low for these workers to consider this employment opportunity. Enhanced layoff risk and exposure to COVID‐19 further deterred respondents, while health insurance, retirement benefits, and a signing bonus enhanced respondents' WTA. The additional unemployment benefits of the CARES Act neither deterred nor encouraged respondents WTA.

## 1 INTRODUCTION

The meatpacking industry has experienced a dearth of workers during the late 2010s and early 2020s (McCracken, 2018; Schutte, 2017). With ever‐present labor pressure and high rates of immigrant workers, an important question is, how do changes in job market conditions (such as a spike in unemployment rates and job‐related health risk) and job‐specific attributes (such as health insurance, retirement benefits, and a signing bonus) impact low‐skilled domestic workers' willingness to accept meatpacking jobs? This study aims to answer this question by exploiting the rapid changes in labor‐market conditions caused by the COVID‐19 pandemic.

The easy and rapid transmission of the novel coronavirus caused many service industry businesses to shutter, leading to historically high unemployment rates. The agricultural and food industries were hit particularly hard because the rapid shutdown of the food service industry caused sudden shifts in demand patterns^1^ and shoulder‐to‐shoulder working conditions, leading to severe outbreaks among workers (Luckstead et al., 2021). With over 500,000 workers and abnormally high infection rates because of difficulties implementing social distancing measures, the meatpacking industry was at the center of employment‐caused outbreaks in the food industry (Cromartie et al., 2020). COVID‐19 outbreaks among workers, changes to worker‐safety rules, shifts in demand patterns, and decline in chicken, eggs, and market hogs (Associated Press, 2020; CDC, 2021a; Kevany, 2020)^2^ caused a reduction in meat supply and variety and also caused price spikes (Bomey & Tyko, 2020; Gellerman, 2020; Repko & Lucas, 2020) because meatpacking facilities shutdown due to a lack of workers as COVID‐19 infections exploded at a time when layoff and unemployment rates were at historic highs (Luckstead et al., 2021). Given the national importance of the meat supply, this study examines low‐skilled workers' willingness to accept meatpacking jobs during the COVID‐19 pandemic. This study examines incentives the meatpacking industry could have used to attract laid off and unemployed workers.

Even with many meatpacking plants located in isolated rural areas, cramped working conditions resulted in the virus spreading rapidly among production‐line workers (Payne, 2020). For instance, the infection rate (2‐week moving average number of new daily cases) in counties with meatpacking operations peaked at just under 50 per 100,000 at the end of April 2020, approximately 10 times higher than rural counties without these operations (Cromartie et al., 2020; Keith Good, 2020; Krisberg, 2020).^3^ By the end of May, the infection rates in counties with meatpacking facilities fell to only seven times that of rural counties without these facilities (Cromartie et al., 2020). Three months later, with new social distancing measures at meatpacking facilities, the new cases in rural counties appeared to be independent of meatpacking facilities. That is, the data show that by the end of August, nonmetro counties with meatpacking facilities experienced similar rates of change as nonmetro counties without meatpacking facilities, although the infection rates in meatpacking‐dependent counties remained approximately 25% higher than other rural counties (Cromartie et al., 2020). Overall, as of February 2021, approximately 270 meatpacking workers had died and 54,000 had contracted the virus, and these numbers are likely underestimated (Abrams, 2021; Bagenstose et al., 2021). The COVID‐19 pandemic has emphasized the important role of meatpacking workers in an effective food supply chain.

Due to unprecedented layoffs and unemployment rates, the US government provided a federal boost of $600 a week and extension to employment benefits through federal pandemic unemployment compensation (FPUC) program as part of the Coronavirus Aid, Relief, and Economic Security (CARES) act. The FPUC policy under the CARES Act expired on July 31, 2020, but the Continued Assistance for Unemployed Workers Act extended the FPUC benefit through March 14, 2021, although it reduced the federal payment to $300 a week (Garner, 2021). Economists estimate that the $600 and $300 supplement resulted in 76% and 48% of workers earning more than their previous income (Chen & Chaney, 2021), respectively. However, further research suggests that the FPUC has not impacted workers' employment search (Bartik et al., 2020; Dube, 2021; Finamor & Scott, 2021; Marinescu et al., 2020). In contrast to the theoretical prediction of a standard job‐search model, Ganong et al. (2021) showed a stable job‐search rate after the heightened replacement rate, and overall, employment was only 0.2%–0.4% lower due to FPUC. By contrast, Fang et al. (2020) estimated that while unemployment insurance expansion under the CARES Act increased the average unemployment rate by 3.8%, FPUC gave workers more flexibility to stay home, which potentially led to a decline in cumulative COVID‐19‐related deaths by 4.9%. Meatpacking was deemed an essential industry and was ordered to remain open (Telford, 2020). However, the rapid spread of COVID‐19 infections made many people sick and unable to work or unwilling to work over fears of catching the virus. This exacerbated the already‐existing dearth of workers in the meatpacking industry and caused this essential industry to operate below full capacity, leading to a fall in meat supply and variety. In addition, high layoff rates nationally and in other low‐skilled industries could result in workers looking to meatpacking for employment.

In nonagricultural sectors, the economics literature has examined the impact of various attributes on the willingness to accept (WTA) job offers (Abraham et al., 2013, considered wages, job quality, and distance from one's home, Cable and Graham (2000) examined communication quality, Jennings et al. (2003) analyzed quality of communication of traditional benefits versus nontraditional benefits for college graduates, and Noe and Barber (1993) studied geographical location). This paper builds on this literature by examining the WTA job offers in an essential industry amid a pandemic.

Studies have also examined the impact of the COVID‐19 pandemic on price dynamics and the supply chain of specific US and Canadian dairy, poultry, beef, pork, and egg segments (Hayes et al., 2021; Lusk et al., 2021; McEwan et al., 2020; Weersink et al., 2020); on exports of grains compared to meat (Mallory, 2020); and on Chinese hog‐pork market (Wang et al., 2020). Rude (2020) and Luckstead and Devadoss (2021) implemented simulation models to examine the impact of COVID‐19‐induced labor shortages and income shocks on the poultry supply chain and cattle and beef industry, respectively. This study relates to Luckstead et al. (2020), who utilized a discrete choice experiment (DCE) implemented before and after the COVID‐19 pandemic to analyze the impact of the pandemic on US domestic workers' WTA temporary agricultural field jobs.^4^ The results show that while domestic workers' reservation wage was well above the wage of a typical field job, the COVID‐19 pandemic and non‐pecuniary benefits (e.g., health insurance, housing, food and clothing allowance, and transportation services) enhance WTA. The current paper builds on this study by studying the impact of the COVID‐19 pandemic, the government response, and enhanced job attributes on the potential labor pool (low‐skilled workers) in an industry (meatpacking) that experienced severe COVID‐19 outbreak.

This study contributes to the literature by implementing a DCE to examine respondents' willingness to make wage concessions as job market conditions (layoff risk, COVID‐19 exposure risk, and extended unemployment benefits) and job‐specific attributes (health insurance, retirement benefits, and a signing bonus) change. In addition, we examine the impact of an information set detailing the effects of COVID‐19 on the meatpacking industry by randomly assigning respondents into a group without this information and a group exposed to this information set. In doing so, this study provides valuable insights for policy makers and industry leaders on the sensitivity of low‐skilled workers to key job attributes, health risk, and government‐enhanced unemployment benefits.

## 2 SURVEY DESIGN AND DATA

We designed an attribute‐based discrete choice experiment to elicit low‐skilled US domestic workers' WTA meatpacking jobs amid the COVID‐19 pandemic and to quantify the perceived monetary equivalent value of heightened unemployment risk, COVID‐19 exposure risk, and other important job attributes (health insurance, retirement benefits, and a signing bonus). We also evaluate the impact of additional information, expanded unemployment benefits under FPUC, and other key questions on the above perceived monetary equivalent values.

Since this study examines whether the COVID‐19‐induced unemployment expanded the potential US meatpacking labor pool, the online survey was limited to respondents residing in the United States and administered via Dynata to generate a sample that is representative and balanced across country demographics (Lorch et al., 2010).^5^ The survey was also restricted to respondents most likely to consider employment in meatpacking facilities by screening out subjects that earned over $75,000 per year, exceeded the retirement age of 65, earned a college degree (BS, BA, PhD, EDD, MD, or Master's degree), or were physically unable to lift 10 pounds or more. As a data quality check, the survey also screened out respondents who appeared inattentive and who spent fewer than 5 and 7 s on the instruction and information pages, respectively (discussed in detail below).

Initially, an optimal orthogonal design was administered to 35 respondents (each answering 8 choice tasks). The resulting estimates of a multinomial logistic model were utilized as Bayesian prior values to generate an efficient Bayesian design in Ngene. This initial pretest sample was excluded from the final data that were used to generate the results in the remainder of the paper. Based on the pretest results, the final survey consisted of three blocks of eight choice tasks, producing a D‐error of 0.0015.

The survey was administered from October 26, 2020, to November 24, 2020, amid the pandemic. This time frame was after the second spike (in terms of daily new COVID‐19 infections) that peaked in late July and at the start of the third, and most severe, spike in the United States (CDC, 2021b). The survey randomly assigned 537 respondents to a control group and 527 respondents to a treated group, for a total number of respondents of 1064. With 1064 respondents and 8 choice tasks, our sample size is 8512. Before the choice task part of the survey, the treatment group was provided the below additional information set that conveys added risk of taking an essential job:

Due to crowded working environments and difficulty in maintaining social distancing in indoor work environments, COVID‐19 infected many meat processing workers. Meat processing facilities addressed the added risk faced by their workers by quarantining work crews infected with coronavirus and shutting down if an outbreak occurred. Executive orders by President Trump required meat packing plants to remain operating, even as many workers were unable to work due to illness or quarantine orders. Nevertheless, because of outbreaks among both workers and management, many meat processing plants temporarily closed or operated at below capacity due to the pandemic. Workers in meat processing plants however are deemed essential amid COVID‐19 since a shortage of workers could significantly affect retail meat supply in the country.

With this treatment group, we can assess the impact of this information on subjects' reservation wage rate and WTA meatpacking jobs amid a pandemic. While there was a flood of information regarding COVID‐19 at the time of the survey, this information set provided information specific to meatpacking. Note that this information set is not required to identify our primary research objective of evaluating unemployment and COVID‐19 exposure risk, health insurance, retirement benefits, and a signing bonus on low‐skilled workers' WTA a meatpacking job.

Respondents also underwent three steps in the survey before they completed the choice tasks to enhance data quality. First, materials on how to complete the choice task questions were provided. Second, a cheap talk script was provided immediately before the choice tasks to potentially mitigate hypothetical bias, given the stated preference nature of the study (see Supporting Information Appendix A).^6^ Third, to ensure that all respondents considered the same meatpacking job, the survey included the following job summary based on real‐world job postings collected from ziprecruiter.com and indeed.com in September 2020 when searching for “meatpacking job”:

The duties required of meat packers include: loading materials and products into package‐processing equipment measuring, weighing and counting product materials moving or lifting heavy objects loading, unloading or stacking products or materials maintaining a clean and safe work area sorting manufacturing materials or products operating packaging machine equipment recording product, packaging and order information on forms and records.

Assume the processing plant jobs differ ONLY by the attributes specified below. All other aspects of the three jobs should be considered the same.

**Figure 1:**
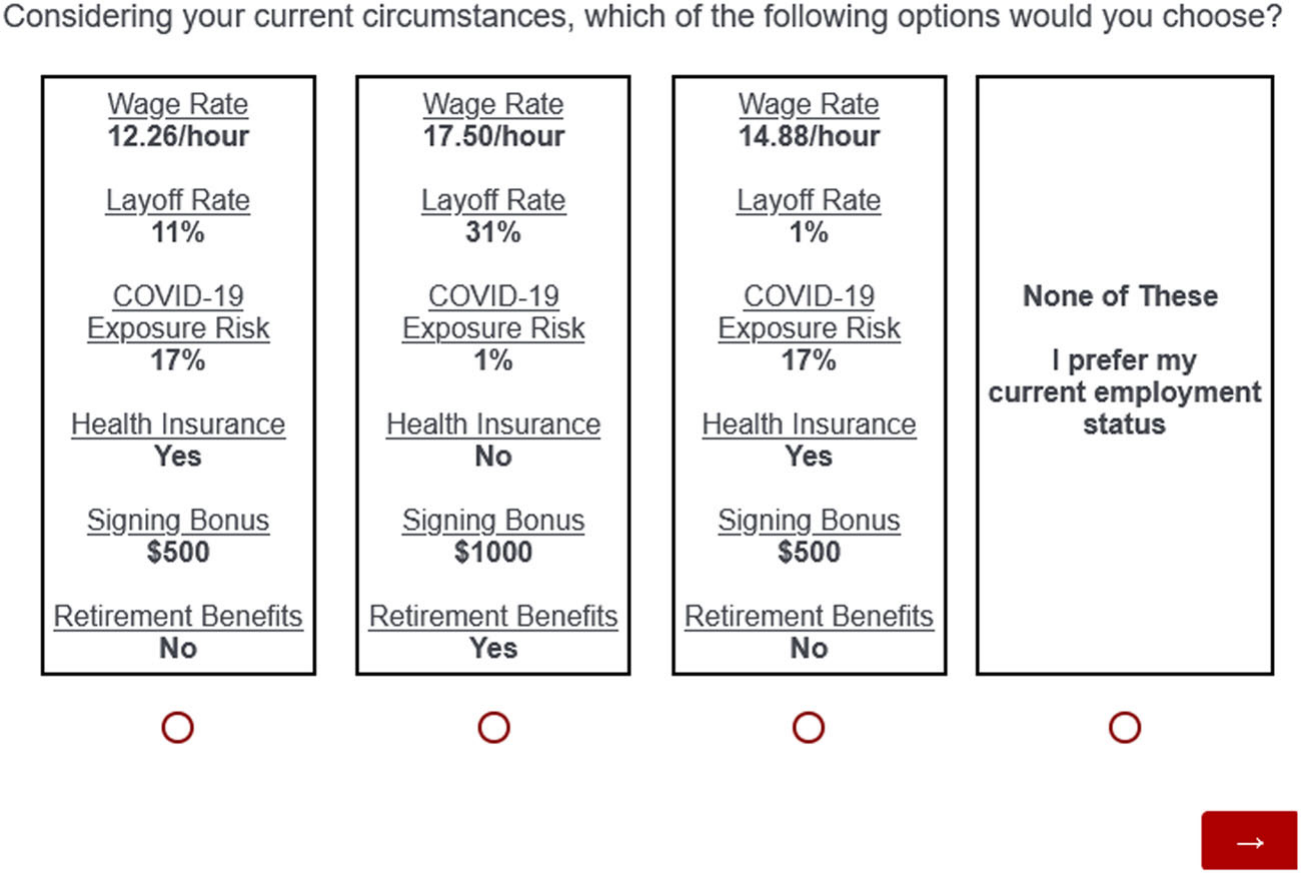
Sample choice tasks from the online survey

Each choice task consisted of three meat processing plant job offers and a “neither of these” option. Based on real‐world job announcements and data on layoffs and COVID‐19 exposure risk at the height of the COVID‐19 pandemic, each job offer included six attributes. As presented in Table 1, the six attributes and corresponding levels are (i) wage rates at $9.64, $12.26, $14.88, and $17.50/h;^7^ (ii) layoff risk of 1%, 11%, 21%, and 31%;^8^ (iii) COVID‐19 exposure risk of 1%, 9%, 17%, and 25%;^9^ (iv) health insurance is a binary yes or no; (v) retirement benefits are a binary yes or no;^10^ and (vi) a signing bonus of $0, $500, and $1000. Our study isolates the impacts of the above six attributes, holding all else equal,^11^ to focus on the impact of these wage and non‐pecuniary wages attributes and pandemic‐induced labor market attributes on WTA job offers in a specific food industry. An example choice set from the online survey is given in Figure 1.

**Table 1:** Attributes and levels of job offer

Wage rate	Layoff risk	COVID‐19 exp. risk	Signing bonus	Health Ins.	Retire. benefit
4 Levels	4 Levels	4 Levels	3 Levels	2 Levels	2 Levels
$9.64/h	1%	1%	$0	Yes	Yes
$12.26/h	11%	9%	$500	No	No
$14.88/h	21%	17%	$1000		
$17.50/h	31%	25%			

The survey also asks questions on race, age, gender, income level, employment status, and previous field work experience to help discern what demographics impact respondents' WTA a meatpacking job. To help determine the driving forces of respondents' WTA a meatpacking job and to assess the impact of expanded FPUC unemployment insurance, the survey incorporated six questions related to health, the CARES Act, and the employment environment:

(1) Are you currently receiving, or did you receive unemployment benefits during the COVID‐19 pandemic? (2) Through the Coronavirus Aid, Relief, and Economic Security (CARES) Act, did you receive (i) The 13 week benefit extension, (ii) The additional $600 per week, or (iii) No additional benefits? Are you or a family member immunocompromised? (3) Are you currently receiving, or did you receive unemployment benefits during the COVID‐19 pandemic? (4) Between March to May of the pandemic what do you believe the probability is of getting a desirable, high paying job? (5) At this point in time [the period the survey was administered], what do you believe the probability is of getting a desirable, high paying job? (6) What do you believe the probability is of getting a desirable, high paying job in January 2021?

Table 2 provides summary statistics for the respondents. An average income of 3.6 indicates that respondents' income on average ranged between $25k and $50k, which is consistent with the average annual salary of $31,671. The average employment status and education values are 1.4 and 3.4, respectively, suggesting that most respondents were either full‐ or part‐time employees and have earned a technical or vocational degree. The respondents were equally split between male and female. And the average household has two adults over the age of 18, roughly 1 child, an hourly wage of $20.7, and works 37.6 h per week. Of the 1064 respondents, 74.9% or 797 reported collecting unemployment and 28.5% or 303 reported that themself or a family member as being immunocompromised. Finally, respondents outlook on getting a desirable well‐paying job improved from 29.5% during the pandemic, to 36.6% during the survey, and to 46.3% looking forward.

**Table 2:** Summary statistics for respondents

	Mean	Std. dev.	Min	Max
Income^a^	3.594	1.728	1	6
Employment status^b^	1.438	0.723	1	4
Education^c^	3.444	1.272	1	5
Gender^d^	0.506	0.500	0	1
No. adults	2.091	1.073	1	7
No. children	0.610	1.015	0	6
Hourly wage rate	20.769	9.882	8	50
Annual salary	31,671.310	21,218.650	0	75,000
Hours per week	37.600	12.994	0	80
Immunocompromised^e^	3.390	1.034	1	4
Unemployment benefits^f^	0.749	0.434	0	1
Unemployed perception^g^				
Prob. during pandemic	29.538	26.039	0	100
Prob. at time of survey	36.559	28.644	0	100
Prob. future (Jan. 2021)	46.258	30.470	0	100

a Five levels: 1, $0–$12,2500 per year; 2, $12,501–$25,000 per year; 3, $25,001–$37,500 per year; 4, $37,501–$49,999 per year; 5, more than $50,000.

b Four levels: 1, employed full time; 2, employed part time; 3, unemployed looking for work; 4, unemployed not looking for work.

c Five levels: 1, primary school/grade school; 2, high school/GED; 3, career school (technical or vocational school) with degree or certification awarded; 4, higher education (community or junior college) with degree or certification awarded; 5, bachelor's degree at university.

^d^ Two levels: 1, male; 0, female.

^e^ Four levels: 1, I am; 2, a family member is; 3, I am and a family member is; 4, none of the above.

^f^ Two levels: 0, yes and 1, no.

^g^ Respondents who identified as unemployed indicated the probability (0% to 100%) of their perception of obtaining a desirable, high‐paying job.

## 3 ECONOMETRIC METHODS

To analyze the survey data, we implement attribute‐based discrete choice methods (Lancaster, 1966; McFadden, 1974). A rational worker considers a bundle of six attributes of a meatpacking job to derive utility. The worker is more likely to accept the employment offer if the combination of attributes leads to the highest utility derived among all employment alternatives. Based on job attributes (A), the utility (U) that individual n obtains from job offer i from a finite set of I alternatives in situation t is

$$U_{nit}=\alpha \prime A_{nit}+\varepsilon_{nit},$$

where α′ is a parameter vector and $\varepsilon_{nit}$ is the random error term. The analysis utilized a random parameters logit (RPL) model because of preference heterogeneity and the breakdown of the Independence from Irrelevant Alternatives property (Train, 2009). Therefore, while RPL models allow for random taste variation, they do not force proportional substitution patterns across alternatives. Furthermore, analysts can model unobserved factors as correlated over time (Train, 2009). Furthermore, RPL allows the analyst to model the panel structure of the data.

The job attribute vector $\left(A_{nit}\right)$ consists of one of four wage rates $\left(W_{it}\right)$, one of four COVID‐19 exposure risk $\left(C_{it}\right)$, one of four layoff risks $\left(L_{it}\right)$, whether or not health insurance $\left(H_{it}\right)$ and retirement $\left(R_{it}\right)$ benefits are included, and one of three signing bonus $\left(B_{it}\right)$. For the RPL model, utility is specified as

$$U_{nit}=ASC_{n}+\alpha_{W}W_{it}+\alpha_{C,n}C_{it}+\alpha_{L,n}L_{it}+\alpha_{H,n}H_{it}+\alpha_{R,n}R_{it}+\alpha_{B,n}B_{it}+\Phi \eta_{n}.$$

$ASC_{n}$ is a binary variable that equals one when respondent n chooses one of the three job alternatives and zero when the respondent chooses the “Neither of These I prefer my current employment status” from the choice set. Thus, $ASC_{n}$ captures preferences that are intrinsic and independent of the explicit attributes. Φ is the lower triangular matrix of the Cholesky decomposition. And, $\eta_{n}$ is distributed multivariate standard normal. The attribute values of utility for the status quo alternative were set to zero for unemployed respondents and set to the self‐reported attribute values for employed respondents.

Using the estimated wage coefficient, $\alpha_{W}$, and the nonwage attribute coefficients, the marginal WTA for an individual product attribute a∈(L,C,B,H,R) is $WTA_{a}=\frac{\alpha_{a}}{\alpha_{W}}$. Because dividing two random parameters could result in infinite moments, we fix the wage while allowing all nonwage attributes a to be normally distributed (Revelt & Train, 1999). Consequently, $WTA_{a}$'s distribution is normal.

## 4 WILLINGNESS TO ACCEPT MEATPACKING JOBS

Next, we present the results of the discrete choice experiment. Table 3 presents the mean WTA a meatpacking job based on RPL regression in the preference space for the full sample with information set interactions and two subsamples for respondents who did not view the additional information set and those that did view the additional information set. The mean WTA values are approximately equal to the ratio of a given attribute to the wage variable in the full RPL regression (provided in Supporting Information Appendix C).^12^ Negative mean WTA values indicate an unwillingness to accept the job offer and the monetary compensation (in $/h) required on average for respondents to be indifferent between accepting a meatpacking job and the respondents' status quo. Positive mean WTA values indicate a willingness to accept the job offer and the amount of money (in $/h) respondents would be willing to give up. For context, it is important to note that according to the summary statistics in Table 2, on average, respondents are employed (the mean employment status is 1.4), paid hourly (mean payment type is 1.705), and worked 37 h a week at a mean wage of $20.769/h.^13^ Therefore, for the average respondent, the status quo is employed with an hourly wage of $20.769/h. The ASC represents the wage adjustment needed for respondents to be indifferent between the job offer and their status quo without any additional job attributes. For the full sample (row 2 of Table 3), the results show that, on average, respondents preferred their current employment situation to employment in a meatpacking facility. Specifically, respondents would require an additional wage of $2.857/h to be indifferent between their status quo and the meatpacking job offer.

**Table 3:** Mean willingness to accept a meatpacking job

	Full sample	Subsamples	
		Information	No information
ASC	−2.857***	0.552	−2.425***
	(0.401)	(0.434)	(0.418)
Layoff	−0.156***	−0.163***	−0.151***
	(0.009)	(0.010)	(0.009)
Exposure	−0.221***	−0.207***	−0.225***
	(0.010)	(0.010)	(0.010)
Insurance	4.564***	3.943***	4.708***
	(0.308)	(0.329)	(0.331)
Retirement	3.427***	3.142***	3.531***
	(0.292)	(0.318)	(0.321)
Bonus	$2.9\times 10^{-3}$ ***	$2.3\times 10^{-3}$ ***	$3.0\times 10^{-3}$ ***
	(0.000)	(0.000)	(0.000)
ASC‐Info	1.381**		
	(0.513)		
Wage‐Info	−0.070*		
	(0.033)		
Layoff‐Info	−0.006		
	(0.011)		
Exposure‐Info	0.010		
	(0.012)		
Insurance‐Info	−0.636*		
	(0.340)		
Retirement‐Info	−0.276		
	(0.335)		
Bonus‐Info	−0.001*		
	(0.000)		
No. obs	8512	4296	4216

*Note*: Standard errors are in given parenthesis. The sample contained 1064 respondents, each which answered eight choice sets, yielding a sample size of 8512 for the full sample. Variables ending with “‐Info” indicate interaction between the attribute and an indicator variable equal to 1 if the respondent viewed the information set and 0 otherwise.

*** p<0.01;

** p<0.05;

* p<0.1.

As expected, with a negative mean WTA, a heightened risk of being laid off and being exposed to COVID‐19 made the meatpacking job offer less desirable. The results show that exposure to COVID‐19 had a larger negative impact on mean WTA than the layoff risk because for each 1% increase in the layoff rate and risk of exposure to COVID, respondents would require an additional $0.156 and $0.221/h, respectively. Therefore, a jump in the unemployment rate of 4%^14^ would require an additional $0.624/h to compensate for the added risk of being laid off. With a national average COVID‐19 exposure rate of 14% at the peak of the pandemic,^15^ workers would require an additional $3.23/h to compensate for the additional risk.

Health insurance, retirement benefits, and a signing bonus are desirable job attributes, as they enhanced workers' willingness to accept meatpacking jobs. The results show that respondents, on average, found health insurance more valuable than a retirement benefit, as the former raises the mean WTA by $4.564/h, while the latter increases the mean WTA by only $3.427/h. Therefore, with an average wage of $20.769/h, respondents would consider a wage of $16.21/h or $17.34/h at a meatpacking facility if health insurance or retirement benefits were part of the employment offer, which is close to the national average hourly wage of $15/h for slaughterers and meat packers in May 2020 (BLS, 2019). Assuming that respondents work 37 h a week for 52 weeks a year, these results imply that respondents are willing to forgo $8781.14 per year or $6593.55 for the opportunity to have health insurance or retirement benefits. For health insurance, this annual reduction in income does not fully cover the 2019 annual Affordable Care Act premium of about $18,240 per year (Goodnough, 2019). Additionally, the retirement benefit implies a wage reduction of 16.5% $\left(=100\times ({\;}^{3.427}{∕}_{20.769}-1)\right)$, which is well above standard employer matching rates of approximately 5%. This suggests that the respondents recognize and account for the long‐term future benefits of retirement programs and are willing to forgo income today to have a better retirement in the future.

Respondents found the signing bonuses to be a desirable job attribute. For example, for every dollar increase in the signing bonus, respondents would be willing to accept a wage reduction of $0.0029/h wage. Therefore, a $500 and $1000 signing bonus, the most commonly seen values in meatpacking job announcements during the pandemic, would have led to a wage reduction of only $1.45 and $2.9/h.^16^

The interaction of the information set with the key job attributes yields interesting results. For example, with a mean WTA value of $1.381, exposure to the information set that detailed the impact of the COVID‐19 pandemic on the meatpacking industry enhanced the willingness to accept meatpacking jobs. Furthermore, the impact of wage rate, health insurance, and a signing bonus on mean WTA decreased as a result of the information set by $0.07, $0.686, and $0.001/h. However, the additional information set did not influence the impact of the layoff rate, exposure risk, or retirement benefit on respondents' WTA, as these results were not statistically significant at the 10% level. Given the pervasive coverage of COVID‐19 news at the time of the survey, these estimates can serve as a lower bound on the impact of this information on the impacts of COVID‐19 on the meatpacking industry.

Next, we examine the impacts of job attributes on WTA a meatpacking job by analyzing subsamples based on respondents that were and were not provided the information set. The impact of job attributes on mean WTA now depends on the subsample being analyzed. For example, respondents who viewed the information set were indifferent between the status quo and the job offer, as the coefficient estimate for ASC is statistically insignificant. By contrast, respondents that did not view the information set preferred their current employment situation and would require a wage boost of $2.425/h (which is similar in magnitude to that in the full sample) to consider a meatpacking job. However, for layoff risk and exposure, the information set did not have differential impacts on the WTA values, as the mean WTA magnitudes in these two subsamples are similar. This finding is consistent with those from the full sample with insignificant interaction terms for layoff risk and exposure. Relative to the full‐sample results, for respondents who viewed the information set, health insurance and retirement bonus attributes were slightly less effective at enhancing workers' WTA, whereas for those who did not view the information set, these attributes were more effective in enhancing workers' WTA a meatpacking job.

From a policy perspective, the US government could have eased the COVID‐19‐induced labor shortage in the meatpacking industry by implementing targeted advertising informing workers of the dire situation of the industry. The results indicate that higher wages along with additional nonwage benefits would have expanded the labor supply. A simple and feasible prescription that would lower respondents' average status quo wage of $22.77/h to the national average hourly wage for slaughterers and meat packers of $15/h (BLS, 2019), a simple remedy is a $3600 signing bonus, which would lead to a wage of $15.2/h (=22.77+2.875−0.0029×3600).^17^ With meatpacking companies offering signing bonuses of between $500 and $1000, government assistance may have been required to achieve this outcome. Finally, policies targeted at limiting the risk of COVID‐19 exposure would mitigate the number of workers leaving this crucial industry.

### 4.1 Subsample analysis

We also conducted subsample analysis based on responses to pandemic‐ and employment‐related questions included in the survey (see Supporting Information Appendix B for the results and a detailed discussion). The results indicate that the respondents who collected unemployment benefits during the pandemic would have preferred a meatpacking job relative to their status quo, and unemployment benefits did not deter people from searching for a job during the COVID‐19 pandemic (Supporting Information Appendix B.1). Interestingly, the additional benefits of the CARES Act neither deterred nor encouraged respondents to accept meatpacking jobs. Furthermore, the results show that immunocompromised workers that are unemployed are highly adverse to leaving their status quo (unemployment) to accept meatpacking jobs (Supporting Information Appendices B.1 and B.3). Thus, it is likely that their unemployment status is a result of their health risk, which contributes to their unwillingness to accept the meatpacking job. However, the relatively small subsample of workers who were unemployed at the time of the survey were also highly adverse to meatpacking jobs (Supporting Information Appendices B.2 and B.3). Since these respondents had a relatively better outlook on future employment, they could have been holding out for better employment opportunities.

## 5 CONCLUSION

This study implemented a discrete choice experiment during the pandemic to examine the impact of the COVID‐19 exposure risk, unemployment risk, enhanced and extended unemployment benefits, and job attributes on the potential labor pool in an industry (meatpacking) that experienced severe COVID‐19 outbreaks. The results of this study provide valuable information for policy makers and industry leaders on mechanisms to alleviate labor shortages for essential food industries during a global pandemic. The result suggests that, in general, respondents were unwilling to accept meatpacking jobs and required a wage premium of $2.857/h to consider a meatpacking job. Enhanced layoff risk and exposure to COVID‐19 further deterred workers from accepting a meatpacking job, while health insurance, retirement benefits, and a signing bonus all enhanced respondents' willingness to accept one. However, an information set on the impact of COVID‐19 on the meatpacking industry resulted in respondents being indifferent between their status quo and the job offer. The mean WTA for layoff rate and risk of exposure are fairly consistent across the various subsamples examined.

The results for the signing bonus suggest a simple policy prescription for easing labor shortages in the meatpacking industry during a pandemic. With a baseline mean WTA of −$2.857/h, a mean WTA $0.0029/h for every $1 increase in the signing bonus, and an average status quo wage of $22.77/h, a $3600 signing bonus would result in a wage of $15.2/h (=22.77+2.875−0.0029×3600), which is very close to the national average hourly wage for slaughterers and meat packers of $15/h (BLS, 2019). It is worth noting that retention of new workers that are naive to the realities of employment in the meatpacking industry is a potential issue that is outside the scope of the current study. Furthermore, counter to expectations for “normal” economic conditions during the COVID‐19 pandemic, respondents who collected unemployment benefits during the pandemic would have preferred a meatpacking job relative to their status quo. In addition, the additional unemployment benefits of the CARES Act neither deterred nor encouraged respondents to consider meatpacking jobs.

## CONFLICTS OF INTEREST

The authors declare no conflicts of interest.

## Supporting information

Supplementary Information

## Data Availability

Data are available on request from the authors.

## Footnotes

1 For instance, as reported by Newman and Bunge (2020), demand for agricultural commodities, such as fresh vegetable and milk, by the food service industry (dine‐in and fast food restaurants, caterers, etc.) plummeted due to stay‐at‐home orders and social distancing measures, which caused a sudden spike in demand for agricultural commodities by food retailers (food stores, food banks, etc.).

2 According to USDA (2020a, 2021a), pig deaths, defined as deaths after weaning, increase by only 0.18% or 22,000 between 2018 and 2019 compared to an expansion of 14% or 1.7 million between 2019 and 2020. Caution must be taken in interpreting this 14% increase as an PRRS 1‐4‐4 outbreak also occurred in 2020 making it impossible to distinguish between pig deaths due to COVID‐19‐induced depopulation and PRRS 1‐4‐4. In terms of poultry, USDA (2020b, 2021b) reported that egg production (both hatching and table eggs) expanded by 2.9% or 3.1 million eggs between 2018 and 2019 but declined by 1.4% or 1.6 million eggs between 2019 and 2020, indicating a reduction in both table eggs and broiler chickens.

3 Over 85% of COVID‐19 infections occurred among minority groups (Bagenstose et al., 2021).

4 See Bernasek and Kinnear (1999), Connelly and Gallagher (2004), and De Cuyper et al. (2008) for related literature on contingent employment.

5 To obtain representative and balanced sample, Dynata “… optimally blend our proprietary sample sources by conducting comparability tests and modeling the blend that will achieve the closest match to census and social benchmarks” (Dynata, 2020). Low‐income respondent may live large distances from a meatpacking plant and confound their willingness to accept a meatpacking job with the distance. We therefore did not include information as to the distance to the meatpacking plant in the survey.

6 See Lusk (2003) and Silva et al. (2011) for discussions on the impacts of a “Cheap Talk” statement on hypothetical bias in attribute‐based DCE.

7 The range of $9.64 to $17.50/h is based on the Occupational Employment Statistics for Food Processing Workers report (BLS, 2019) and job announcements collected in September of 2020.

8 The layoff risk range of 1% to 31% is based on national, manufacturing, leisure and hospitality, and construction statistics for February 2020, before COVID‐19 disseminated throughout the economy, and March and April, the peak of COVID‐19 pandemic‐induced unemployment, from the May and June Job Openings and Labor Turnover reports (BLS, 2020a, 2020b). We include layoff risk from several sectors because respondents considering meatpacking jobs may consider layoff risk from a variety of sectors when considering employment in meatpacking.

9 These exposure rates are based on the CDC's Morbidity and Mortality Weekly Report, COVID‐19 Among Workers in Meat and Poultry Processing Facilities (Waltenburg et al., 2020).

10 The health insurance, signing bonus, and retirement benefits are based on job announcements collected in August and September of 2020 for meatpacking production workers.

11 Job attributes that are not considered non‐pecuniary wages and thus outside the control of employers (that we hold constant but could impact consumers WTA a meatpacking job) include distance and prestige of the job.

12 The income for salaried workers and for those collecting unemployment benefits were converted to $/h based on standard hours worked in a year or week to maintain consistency in the wage variable.

13 The average respondent lived in a suburban area, and of all respondents, 57% were White, 22% were Black or African American, 12% were Hispanic or Latino, and 89.5% were born in the United States.

14 BLS (2020a) reports that turnover (quits, layoffs and discharges, and other separations) increased from 3.7% in May 2019% to 7.6% in April 2021 nationally.

15 Waltenburg et al. (2020) report that between April and May 2020, COVID‐19 impacted 121,616 workers in meat/poultry facilities, with 16,233 positive COVID‐19 tests.

16 Each respondent was asked what attributed they ignored during the choice task; the attributes (with the number of respondents that selected it in parentheses) are Wage Rate (50), Layoff Rate (174), COVID‐19 Exposure Risk (158), Health Insurance (99), Signing Bonus (188), Retirement Benefits (140), None ‐ I paid attention to all aspects (589); note that the numbers add up to more than 1064 because respondents could select more than one option. Of the 344 respondents that selected the “neither of these” option in the choice task section of the survey, 93 indicated the “Pay was too low,” 76 indicated “I don't want a meat processing job,” 111 indicated “I prefer my current job”, 37 indicated “I believe I can find something better,” and only 4 indicated “I prefer unemployment.”

17 Note that a targeted information campaign aimed at informing workers of the dire situation coupled with a signing bonus of $3,300 would also be sufficient to achieve a wage of $15.18/h (=22.77−0.0023×3300).
