# Supplementary material for: US workers' willingness to accept meatpacking jobs amid the COVID‐19 pandemic
Source: J Agric Appl Econ Asssoc. 2022 Apr 27;1(1):47–60. doi: 10.1002/jaa2.8 (PMC9087796; doi:10.1002/jaa2.8)
Supplement: Supplementary file 1 — Supplementary Information [file JAA2-1-47-s001.pdf]

Appendix for

US Workers' Willingness to Accept Meatpacking Jobs Amid the COVID-19 Pandemic

A Pre-Choice Task Cheap Talk Statement

As nonpartisan researchers, we care about the quality of our survey data and hope to receive the most accurate measures of your opinions. So, it is important that you carefully read all the information provided, and that you thoughtfully give your best answer to each question in the survey. To maintain the integrity of the study, certain measurements have been put in place throughout the survey so as to flag respondents who do not provide quality or complete responses. Do you commit to carefully reading and providing your thoughtful and honest answers to the questions in this survey?

I will read carefully and provide my best answers

I will not read carefully and provide my best answers

I can't promise either way

B Question-Based Subsample Analysis

Next, we consider the impact of subsamples based on responses to pandemic- and employment-related questions included in the survey.

B.1 Unemployment Benefits, CARES, Health

Table 1 reports the mean WTA results for subsamples based on three questions: (i) Are you currently receiving, or did you receive unemployment benefits during the COVID-19

Table 1: Mean WTA for Subsamples: Unemployment, CARES, Health

	Collect Unemployment		CARES		Immunocompromised	
	Yes	No	Yes	No	Yes	No
ASC	3.188**	-0.449	0.222	7.242**	2.479	-0.561
	(1.486)	(0.813)	(1.677)	(3.066)	(1.586)	(0.765)
Layoff	-0.139***	-0.122***	-0.154***	-0.103**	-0.139***	-0.123***
	(0.018)	(0.01)	(0.026)	(0.041)	(0.017)	(0.01)
Exposure	-0.169***	-0.159***	-0.167***	-0.189***	-0.167***	-0.160***
	(0.028)	(0.012)	(0.026)	(0.06)	(0.02)	(0.013)
Insurance	3.355***	3.332***	2.775***	4.188***	2.927***	3.265***
	(0.481)	(0.339)	(0.576)	(1.165)	(0.504)	(0.301)
Retirement	2.259***	2.472***	1.652***	4.213***	2.193***	2.477***
	(0.413)	(0.276)	(0.442)	(1.238)	(0.444)	(0.266)
Bonus	4×10^{-4}***	1.7×10^{-3}***	1.1×10^{-3} ***	2×10^{-4}	2×10^{-3}***	7×10^{-4}***
	(1×10^{-4})	(2×10^{-4})	$(4 \times 10^{-4}$)	$(3 \times 10^{-4}$)	(4×10^{-4})	(1×10^{-4})
No Obs	2,136	6,376	1,584	552	2,424	6,088

Notes: Standard errors are in parenthesis. *** $p < 0.01$, ** $p < 0.05$, * $p < 0.1$.

pandemic? (ii) Did the respondent benefit from the CARES act? (iii) Are you or a family member immunocompromised? For respondents that answered these questions, the sample was further broken down based on whether the answer was "yes" or "no".

First, we divide the sample based on whether unemployment benefits were collected at the time of the survey or during the pandemic (2nd and 3rd columns of Table 1). Based on the survey results, 74.9% of respondents indicated that they applied for unemployment starting, on average, in March or April of 2020, with benefits ending in August 2020. Additionally, the average unemployment benefit was \$503/week, although the standard deviation was high at \$335.8/week. The results indicate that the respondents who collected unemployment during the pandemic would have preferred a meatpacking job relative to their status quo. Specifically, for this subset of respondents, the mean WTA was 3.188, implying on average that the respondents would have been indifferent between a \$3.188/hour pay cut and their status quo at the time of the survey. This result also suggests that during the COVID-19 pandemic, unemployment benefits did not deter people from searching for a job. However, respondents that did not collect unemployment were indifferent between their current status and the meatpacking job, as the ASC mean WTA is statistically insignificant. This could

be because respondents in this subsample had satisfactory employment or were relatively satisfied in unemployment without receiving any benefits during the pandemic.

For both subsamples, the results suggest that, with negative mean WTA values similar to those of the main results, as the layoff rate and risk of exposure to COVID-19 increased, respondents' willingness to accept meatpacking job declined. Additionally, while health insurance, retirement benefits, and signing bonuses all enhanced the respondents' WTA, these job attributes were not as effective in attracting workers from this subsample, as the magnitude of the mean WTA is lower than those of the main results in Table 3.

Second, for respondents who answered the question regarding additional benefits from the CARES Act, we divided the subsample into respondents that received the CARES benefit at any point and those that did not receive additional benefits (4th and 5th columns of Table 1). The additional benefits of the CARES Act neither deterred nor encouraged respondents to accept meatpacking jobs because the mean WTA for ASC is not statistically different from zero. This finding is in contrast to the negative mean WTA results of Table 3 and is consistent with the literature that FPUC under the CARES Act did not impact employment gaps amid the pandemic (Dube, 2021; Bartik et al., 2020; Marinescu et al., 2020; Finamor and Scott, 2021).

The mean WTA for layoff rate and risk of exposure are similar in magnitude to those of the main results. Additionally, relative to the main results in Table 3, additional benefits through the CARES act dampened the effect of health insurance, retirement policy, and signing bonuses on enhancing workers' WTA meatpacking jobs between 39% and 62%. Therefore, respondents that received additional benefits under the CARES were less willing to substitute health insurance, retirement policy, and signing bonuses for lower wage rates. These results could be because health insurance and retirement policy do not alleviate of the severe negative income shock that many experienced due to COVID-19 (Chen and Chaney, 2021).

The subset of respondents who did not receive additional benefits under the CARES

Act were the most willing to accept a meatpacking job, as they would have been indifferent between a $7.242/hour pay cut and the status quo at the time of the survey. However, the mean WTA for layoff risk and exposure were again fairly similar in magnitude to the main results. Additionally, as expected, health insurance and retirement benefits enhanced their WTA. However, the signing bonus did not influence this group's decisions, as the mean WTA is insignificant.

Third, we divided the sample into respondents who were and were not immunocompromised (6th and 7th columns of Table 1). A priori, we would have expected immunocompromised respondents to avoid employment that involves a high risk of contracting COVID-19. Therefore, it is counterintuitive that when the sample is divided between respondents that were or had a family member who was immunocompromised, respondents were indifferent between their current status and a meatpacking job (i.e., the estimated mean WTA for ASC is insignificant). However, this result could occur because our sample includes low-income people,[1] which implies that alternative employment opportunities for these respondents might also have a relatively high COVID-19 exposure risk. The results show that the mean WTA for layoff, exposure, insurance, and retirement bonuses for the immunocompromised subsamples are again similar in magnitude to the main results. To provide additional insight into the counterintuitive result, Appendix B, Table 3, we rerun analysis for immunocompromised respondents while including an unemployment interaction. The results indicate that including the unemployment interaction had minimal effects on the results the main attributes (ASC, layoff, exposure, insurance, and bonus) as the mean WTA in Table 3 are similar to those from Table 1 without the interaction. However, the key result is the mean WTA value of -$17.405 for the ASC-Unemp term, which indicates that immunocompromised workers that are unemployed are highly adverse to leaving their status quo (unemployment) to accept meatpacking jobs. Thus, it is likely that their unemployment status is a result of their health risk, which contributes to their unwillingness to accept the meatpacking job.

[1] Annual income was 31,671 on average, with a standard deviation of 21,218.

B.2 Unemployed Workers

Because unemployed workers are the likely to enter the meatpacking industry and expand the labor supply, we consider a subset of 93 respondents that were unemployed and actively looking for work at the time of the survey. With 93 respondents and 8 choice sets, the sample size is 744. Of the 93 respondents in this subsample, 66 were actively looking for work and 27 were not looking for work (see Appendix C for sensitivity analysis where we further limit the sample to unemployed that are actively looking for work). In addition to being unemployed, we asked the respondents to indicate the probability (0% to 100%) of their perception of obtaining a desirable, high-paying job before the survey, at the time of the survey, and in the future. As reported in Table 2, these 93 respondents indicated, on average, a 29.54% change before the survey, which increased to 36.56% at the time of the survey and 46.26% by January 2021. Thus, while respondents believed that the probability of obtaining a desirable, high-paying job was below 50%, their outlook improved over time.

In three separate analyses, we interacted the answers to each of the three job-outlook questions with the job attributes. Table 2 reports the mean WTA for this unemployed subsample. The results for this subgroup are striking—the respondents were considerably less willing to accept a meatpacking job compared to the full sample, as the ASC value is substantially more negative at -15.269, -15.999, and -17.819 for the past, present, and future, respectively, relative to -2.857 for the full sample in Table 3. These results could have occurred because approximately 30% of the subgroup is not looking for work; therefore, a large wage premium would be required for these respondents to enter the workforce in a meatpacking job. Additionally, the respondents who were unemployed but looking for work had a relatively better outlook on future employment at 50.5%, compared to 46.26% for all unemployed workers and 45.36% for employed workers. Therefore, it is possible that these respondents could be holding out for better employment opportunities.

Interacting the job attributes with the respondents' perception (evaluated as a probability from 0% to 100%) of obtaining a desirable, high-paying job influences the mean WTA for

ASC, but only very slightly (0.173, 0.195, 0.159 for past, present, and future, respectively). Again, the impact of layoff and exposure have a very similar impact on mean WTA for "past" and "present," as in the main results in Table 3. However, when the job attributes are interacted with unemployed respondents' future outlook on employment opportunities, the impact of layoffs becomes statistically insignificant, which is consistent with these respondents' improvement in employment outlook over time. The mean WTA for exposure to COVID-19 and insurance are also similar to the main results in Table 3. However, the retirement attribute and bonus are statistically insignificant, which suggests that respondents in this subsample have either left the workforce or are holding out for better jobs. Note that, for the subsample of respondents that were employed at the time of the survey, the mean WTA values are very similar to the main results in Table 3. Given the similarity in the results, we do not report these results in the tables.

B.3 Sensitivity Analysis for Mean WTA

Table 3 reports the results for immunocompromised workers or family members with interactions for unemployed workers. The important result here is mean WTA value of -$17.405 for ASC-Unemp term which indicates that immunocompromised workers that are unemployed are highly adverse to accept meatpacking jobs. This implies that they are unemployed because of their health condition and are not willing to accept the meatpacking job. The only other interaction term that is statistically significant is layoff rate, which suggest that a higher layoff rate has a small positive impact on immunocompromised workers that are unemployed willingness to accept a meat packing job.

Table 4 reports the results for unemployed workers that are actively looking for work. The signs and magnitudes of the coefficient estimates are similar to those from Table 2 in the main text, although the interaction terms are largely insignificant.

Table 2: Mean WTA for Subsamples: Employment and Job Perception

	Unemployed		
	Past	Present	Future
ASC	-15.269***	-15.999***	-17.819***
	(2.788)	(3.612)	(3.573)
Layoff	-0.119**	-0.114**	-0.062
	(0.049)	(0.057)	(0.053)
Exposure	-0.311***	-0.272***	-0.243**
	(0.094)	(0.1)	(0.097)
Insurance	4.238***	4.246**	4.210**
	(1.552)	(1.961)	(1.899)
Retirement	2.009	1.716	1.487
	(1.27)	(1.423)	(1.516)
Bonus	0.001	0.000	0.001
	(0.001)	(0.001)	(0.001)
ASC-Prob	0.173**	0.195**	0.159**
	(0.082)	(0.085)	(0.065)
wage-Prob	-0.007	-0.004	-0.002
	(0.004)	(0.004)	(0.004)
Layoff-Prob	0.002*	0.001	0.000
	(0.001)	(0.001)	(0.001)
Exposure-Prob	0.003*	0.002	0.001
	(0.001)	(0.002)	(0.001)
Insurance-Prob	-0.054**	-0.037	-0.035
	(0.026)	(0.03)	(0.024)
Retirement-Prob	0.004	0.006	0.008
	(0.022)	(0.024)	(0.02)
Bonus-Prob	0 .000	0 .000	0 .000
	(0.001)	(0.001)	(0.001)
No Obs	744	744	744

Notes: Standard errors are in parenthesis. *** $p < 0.01$, ** $p < 0.05$, * $p < 0.1$. Variables ending with "-Prob" indicate interaction between the attribute and an indicator variable equal to one if the respondent viewed the information set and zero otherwise.

Table 3: Mean WTA for Subsamples: Immunocompromised with Unemployment Interactions

	Immunocompromised
	Yes
ASC	2.276
	(1.816)
Layoff	-0.134***
	(0.019)
Exposure	-0.154***
	(0.022)
Insurance	2.734***
	(0.481)
Retirement	2.193***
	(0.431)
Bonus	0.002**
	(0.001)
ASC-Unemp	-17.405***
	(6.262)
Wage-Unemp	-0.296
	(0.279)
Layoff-Unemp	0.091*
	(0.055)
Exposure-Unemp	-0.001
	(0.073)
Insurance-Unemp	1.776
	(1.488)
Retirement-Unemp	-1.317
	(1.378)
Bonus-Unemp	0.001
	(0.001)
No Obs	2,424

Notes: Standard errors are in parenthesis. $^{***}p < 0.01$, $^{**}p < 0.05$, $^{*}p < 0.1$. Variables ending with "-Unemp" and "-Immuno" indicate interaction between the attribute and an indicator variable equal to one if the respondent were unemployed or immunocomprimized, respectively.

Table 4: Mean WTA for Subsamples: Employment and Job Perception

	Unemployed		
	Past	Present	Future
ASC	-15.15***	-11.405***	-17.907***
	(2.726)	(3.577)	(3.046)
Layoff	-0.166**	-0.102**	-0.093
	(0.081)	(0.051)	(0.062)
Exposure	-0.288**	-0.224***	-0.227*
	(0.13)	(0.086)	(0.117)
Insurance	4.377**	3.655**	4.158**
	(1.908)	(1.516)	(2.055)
Retirement	2.124	1.214	1.146
	(1.27)	(1.199)	(1.485)
Bonus	0.001	0.001	0.001
	(0.001)	(0.001)	(0.001)
ASC-Prob	0.09	-0.01	0.113*
	(0.102)	(0.102)	(0.064)
wage-Prob	-0.005	-0.004	-0.002
	(0.006)	(0.004)	(0.005)
Layoff-Prob	0.003	0.001	0.001
	(0.002)	(0.001)	(0.001)
Exposure-Prob	0.003	0.001	0 .001
	(0.002)	(0.002)	(0.002)
Insurance-Prob	-0.062*	-0.036	-0.032
	(0.034)	(0.025)	(0.029)
Retirement-Prob	-0.009	0.013	0.009
	(0.028)	(0.023)	(0.022)
Bonus-Prob	0	0*	0
	(0)	(0)	(0)
No Obs	528	528	528

Notes: Standard errors are in parenthesis. *** $p < 0.01$, ** $p < 0.05$, * $p < 0.1$. Variables ending with "-Prob" indicate interaction between the attribute and an indicator variable equal to one if the respondent viewed the information set and zero otherwise.

C RPL Regression Results

The RPL models are estimated using the "gmnl" package in R (Version 3.4.3) with 500 Halton draws such that the simulation accounts for the panel structure of the data (Sarrias et al., 2017).

References

Bartik, A. W., M. Bertrand, F. Lin, J. Rothstein, and M. Unrath (2020). Measuring the labor market at the onset of the covid-19 crisis. Technical Report WORKING PAPER 27613, National Bureau of Economic Research. DOI: 10.3386/w27613.

Chen, T.-P. and S. Chaney (2021). Laid-Off Workers Cut Spending, Hunt for Jobs as Extra Unemployment Benefits Run Out. The Wall Street Journal. Accessed on February 17, 2021.

Dube, A. (2021). Aggregate employment effects of unemployment benefits during deep downturns: Evidence from the expiration of the federal pandemic unemployment compensation. Technical Report WORKING PAPER 28470, National Bureau of Economic Research. DOI: 10.3386/w2847.

Finamor, L. and D. Scott (2021). Labor market trends and unemployment insurance generosity during the pandemic. *Economics Letters 199*, 109722.

Marinescu, I. E., D. Skandalis, and D. Zhao (2020). Job search, job posting and unemployment insurance during the covid-19 crisis. SSRN.

Sarrias, M., R. Daziano, et al. (2017). Multinomial logit models with continuous and discrete individual heterogeneity in r: the gmnl package. *Journal of Statistical Software 79*(2), 1–46.

Table 5: RPL for Full Sample and Information Set Subsamples

	Full		Information Set		No Information set	
wage	0.2243	0.0071	0.2105	0.0076	0.2269	0.0085
JobASC.Info	0.3098	0.1140	0.1162	0.0925	-0.5503	0.0904
wage.Info	-0.0156	0.0077	-0.0344	0.0021	-0.0341	0.0022
layoff.Info	-0.0013	0.0025	-0.0436	0.0023	-0.0511	0.0025
exposure.Info	0.0023	0.0027	0.8299	0.0675	1.0684	0.0716
insurance.Info	-0.1426	0.0759	0.6614	0.0648	0.8014	0.0720
retirement.Info	-0.0619	0.0751	0.0005	0.0001	0.0007	0.0001
bonus.Info	-0.0002	0.0001	5.6495	0.2190	-6.5013	0.2517
JobASC	-0.6410	0.0852	0.0174	0.0025	-0.0188	0.0026
layoff	-0.0351	0.0020	0.0224	0.0028	-0.0290	0.0029
exposure	-0.0495	0.0022	-0.0952	0.0783	0.3250	0.0796
insurance	1.0238	0.0637	-0.1886	0.0795	0.3534	0.0830
retirement	0.7687	0.0633	-0.0001	0.0001	0.0002	0.0001
bonus	0.0007	0.0001	0.0316	0.0035	0.0309	0.0033
sd.JobASC.JobASC	5.9734	0.1650	0.0035	0.0034	0.0111	0.0032
sd.JobASC.layoff	0.0189	0.0018	-0.2884	0.0972	0.1087	0.1028
sd.JobASC.exposure	0.0257	0.0020	-0.1047	0.0944	0.0237	0.1009
sd.JobASC.insurance	-0.2033	0.0555	-0.0001	0.0001	-0.0003	0.0001
sd.JobASC.bonus	-0.2762	0.0572	0.0405	0.0034	0.0472	0.0032
sd.JobASC.retirement	-0.0001	0.0001	0.1439	0.0925	0.1377	0.0933
sd.layoff.layoff	0.0310	0.0025	0.2857	0.0922	0.1197	0.0923
sd.layoff.exposure	0.0089	0.0024	-0.0003	0.0001	-0.0002	0.0001
sd.layoff.insurance	-0.0726	0.0718	0.7869	0.0995	0.6955	0.1164
sd.layoff.bonus	-0.0847	0.0709	0.2105	0.0942	0.1625	0.1100
sd.layoff.retirement	-0.0002	0.0001	-0.0002	0.0001	0.0001	0.0001
sd.exposure.exposure	0.0435	0.0023	-0.5883	0.1127	0.7520	0.1079
sd.exposure.insurance	0.1130	0.0650	-0.0001	0.0001	-0.0003	0.0001
sd.exposure.bonus	0.2192	0.0646	0.0006	0.0001	0.0006	0.0001
sd.exposure.retirement	-0.0003	0.0001				
sd.insurance.insurance	-0.7573	0.0751				
sd.insurance.bonus	-0.1947	0.0702				
sd.insurance.retirement	0.0001	0.0001				
sd.bonus.bonus	0.6167	0.0804				
sd.bonus.retirement	-0.0001	0.0001				
sd.retirement.retirement	-0.0007	0.0001				

Table 6: RPL Collecting Unemployment

	No		Yes	
Parameter	Estimate	Std. Error	Estimate	Std. Error
bonus	0.0006	0.0001	0.0004	0.0001
JobASC	-0.152	0.2744	0.0742	0.5608
wage	0.3388	0.0177	0.3336	0.0409
layoff	-0.0412	0.0032	-0.0514	0.0076
exposure	-0.0538	0.0041	-0.0556	0.0079
insurance	1.1287	0.0996	0.9256	0.1766
retirement	0.8374	0.0826	0.5509	0.144
L.JobASC.JobASC	6.4459	0.344	7.8426	0.7053
L.JobASC.wage	0.0359	0.0165	0.0186	0.0217
L.JobASC.layoff	0.0149	0.0039	0.0311	0.0071
L.JobASC.exposure	0.0166	0.0047	0.0302	0.0085
L.JobASC.insurance	-0.0471	0.1095	-0.3116	0.2145
L.JobASC.retirement	-0.2747	0.0981	-0.1677	0.1621
L.wage.wage	0.2941	0.0176	0.4164	0.0403
L.wage.layoff	-0.0049	0.0043	-0.0038	0.0063
L.wage.exposure	-0.0167	0.0044	-0.0061	0.007
L.wage.insurance	0.2566	0.1057	0.1794	0.174
L.wage.retirement	0.1184	0.0902	0.1134	0.1626
L.layoff.layoff	0.0388	0.0039	0.0396	0.006
L.layoff.exposure	0.0007	0.0043	-0.0056	0.007
L.layoff.insurance	-0.0213	0.1288	-0.2334	0.217
L.layoff.retirement	0.0319	0.1125	-0.3878	0.1694
L.exposure.exposure	0.052	0.0035	0.0553	0.0077
L.exposure.insurance	0.0375	0.1138	-0.2853	0.261
L.exposure.retirement	0.1227	0.1031	-0.1793	0.2007
L.insurance.insurance	1.2055	0.1003	0.9778	0.1718
L.insurance.retirement	0.289	0.1176	0.1976	0.181
L.retirement.retirement	0.9014	0.0932	-0.7113	0.1789
SD.JobASC	6.4459	0.344	7.8426	0.7053
SD.wage	0.2963	0.0169	0.4168	0.0403
SD.layoff	0.0418	0.0039	0.0505	0.0071
SD.exposure	0.057	0.0037	0.0636	0.0083
SD.insurance	1.2342	0.0998	1.1051	0.1854
SD.retirement	1.0008	0.0948	0.8767	0.1651

Table 7: RPL Benefits Extension through the CARES Act

	No		Yes	
Parameter	Estimate	Std. Error	Estimate	Std. Error
bonus	0	0.0001	0.0004	0.0001
JobASC	1.3199	0.5687	0.0742	0.5608
wage	0.1823	0.0343	0.3336	0.0409
layoff	-0.0188	0.0076	-0.0514	0.0076
exposure	-0.0345	0.0103	-0.0556	0.0079
insurance	0.7632	0.1949	0.9256	0.1766
retirement	0.7678	0.1789	0.5509	0.144
L.JobASC.JobASC	4.1196	0.6672	7.8426	0.7053
L.JobASC.wage	0.0244	0.0367	0.0186	0.0217
L.JobASC.layoff	0.0004	0.0122	0.0311	0.0071
L.JobASC.exposure	0.0363	0.0126	0.0302	0.0085
L.JobASC.insurance	0.1655	0.3065	-0.3116	0.2145
L.JobASC.retirement	-0.0271	0.2401	-0.1677	0.1621
L.wage.wage	0.1959	0.0347	0.4164	0.0403
L.wage.layoff	-0.0219	0.0097	-0.0038	0.0063
L.wage.exposure	-0.0095	0.0129	-0.0061	0.007
L.wage.insurance	0.521	0.2481	0.1794	0.174
L.wage.retirement	0.0587	0.2356	0.1134	0.1626
L.layoff.layoff	-0.0236	0.0107	0.0396	0.006
L.layoff.exposure	0.0092	0.0117	-0.0056	0.007
L.layoff.insurance	0.5952	0.2765	-0.2334	0.217
L.layoff.retirement	0.5022	0.2609	-0.3878	0.1694
L.exposure.exposure	0.0539	0.0143	0.0553	0.0077
L.exposure.insurance	-0.1545	0.3064	-0.2853	0.261
L.exposure.retirement	-0.5132	0.2677	-0.1793	0.2007
L.insurance.insurance	-0.3248	0.3545	0.9778	0.1718
L.insurance.retirement	-0.099	0.2944	0.1976	0.181
L.retirement.retirement	0.212	0.3431	-0.7113	0.1789
SD.JobASC	4.1196	0.6672	7.8426	0.7053
SD.wage	0.1974	0.0345	0.4168	0.0403
SD.layoff	0.0322	0.0091	0.0505	0.0071
SD.exposure	0.0663	0.0144	0.0636	0.0083
SD.insurance	0.8845	0.2495	1.1051	0.1854
SD.retirement	0.7579	0.2355	0.8767	0.1651

Table 8: RPL for Immunocompromised People

	No		Yes	
Parameter	Estimate	Std. Error	Estimate	Std. Error
bonus	0.0003	0	0.0004	0.0001
JobASC	-0.2031	0.2746	0.0742	0.5608
wage	0.3618	0.0201	0.3336	0.0409
layoff	-0.0444	0.0033	-0.0514	0.0076
exposure	-0.0579	0.0041	-0.0556	0.0079
insurance	1.1811	0.0958	0.9256	0.1766
retirement	0.8962	0.0844	0.5509	0.144
L.JobASC.JobASC	7.1183	0.3562	7.8426	0.7053
L.JobASC.wage	0.004	0.0172	0.0186	0.0217
L.JobASC.layoff	0.0171	0.004	0.0311	0.0071
L.JobASC.exposure	0.0244	0.0046	0.0302	0.0085
L.JobASC.insurance	-0.2357	0.1135	-0.3116	0.2145
L.JobASC.retirement	-0.2626	0.1078	-0.1677	0.1621
L.wage.wage	0.3079	0.0167	0.4164	0.0403
L.wage.layoff	-0.0047	0.0036	-0.0038	0.0063
L.wage.exposure	-0.0035	0.0046	-0.0061	0.007
L.wage.insurance	0.2628	0.1048	0.1794	0.174
L.wage.retirement	0.105	0.0937	0.1134	0.1626
L.layoff.layoff	0.0448	0.0047	0.0396	0.006
L.layoff.exposure	0.0095	0.006	-0.0056	0.007
L.layoff.insurance	0.0471	0.1279	-0.2334	0.217
L.layoff.retirement	0.0288	0.1345	-0.3878	0.1694
L.exposure.exposure	0.0612	0.0066	0.0553	0.0077
L.exposure.insurance	0.1323	0.1385	-0.2853	0.261
L.exposure.retirement	0.0198	0.1201	-0.1793	0.2007
L.insurance.insurance	1.1454	0.1073	0.9778	0.1718
L.insurance.retirement	0.1643	0.1156	0.1976	0.181
L.retirement.retirement	0.8537	0.0954	-0.7113	0.1789
SD.JobASC	7.1183	0.3562	7.8426	0.7053
SD.wage	0.308	0.0167	0.4168	0.0403
SD.layoff	0.0482	0.0049	0.0505	0.0071
SD.exposure	0.0666	0.0066	0.0636	0.0083
SD.insurance	1.2067	0.1067	1.1051	0.1854
SD.retirement	0.9148	0.0948	0.8767	0.1651

Table 9: RPL for Unemployed Workers

	Before		Currnet		Future	
Parameter	Estimate	Std. Error	Estimate	Std. Error	Estimate	Std. Error
JobASC.unemp_prob_pan	0.0621	0.037	0.0637	0.0363	0.0547	0.0329
wage.unemp_prob_pan	-0.0023	0.0019	-0.0012	0.0017	-0.0006	0.0017
layoff.unemp_prob_pan	0.0006	0.0003	0.0003	0.0003	-0.0001	0.0003
exposure.unemp_prob_pan	0.0009	0.0005	0.0006	0.0005	0.0002	0.0005
insurance.unemp_prob_pan	-0.0195	0.0077	-0.0121	0.009	-0.0122	0.0076
retirement.unemp_prob_pan	0.0013	0.0079	0.002	0.0079	0.0027	0.007
bonus.unemp_prob_pan	0	0	0	0	0	0
bonus	0.0004	0.0003	0	0.0004	0.0004	0.0004
JobASC	-5.4834	1.8244	-5.2352	1.8933	-6.1371	2.3241
wage	0.3591	0.0999	0.3272	0.093	0.3444	0.117
layoff	-0.0426	0.0139	-0.0373	0.0152	-0.0212	0.0166
exposure	-0.1117	0.0234	-0.0891	0.0257	-0.0837	0.0278
insurance	1.522	0.3655	1.3893	0.4875	1.4501	0.4972
retirement	0.7216	0.362	0.5614	0.4169	0.512	0.456
L.JobASC.JobASC	9.0912	1.4289	-7.5067	1.1349	8.9915	1.4031
L.JobASC.wage	-0.1701	0.0636	0.2454	0.0536	-0.225	0.0654
L.JobASC.layoff	-0.0041	0.0116	0.0092	0.0106	-0.0017	0.0109
L.JobASC.exposure	0.0356	0.0177	0.0012	0.0162	0.0323	0.0182
L.JobASC.insurance	0.1385	0.3335	-0.5092	0.3096	0.2592	0.3357
L.JobASC.retirement	-0.5111	0.2907	-0.0233	0.2807	-0.2358	0.2872
L.wage.wage	0.2401	0.039	0.2794	0.0417	0.226	0.0341
L.wage.layoff	0.0042	0.0097	0.0088	0.0122	0.005	0.0099
L.wage.exposure	0.0015	0.0154	-0.0115	0.0193	-0.0102	0.0227
L.wage.insurance	-0.2863	0.29	-0.0784	0.3173	-0.2081	0.318
L.wage.retirement	-0.8126	0.2585	-0.9426	0.3005	-0.8905	0.2702
L.layoff.layoff	0.0265	0.0099	0.0334	0.0127	0.0309	0.0124
L.layoff.exposure	0.0499	0.0153	0.0559	0.0188	0.0607	0.0234
L.layoff.insurance	-0.3618	0.2705	-0.4822	0.3172	-0.1582	0.2982
L.layoff.retirement	0.1716	0.2421	0.1325	0.2336	0.2499	0.2669
L.exposure.exposure	0.0562	0.0144	-0.0687	0.0179	0.0516	0.0182
L.exposure.insurance	0.489	0.3406	-0.1973	0.274	0.2129	0.3099
L.exposure.retirement	-0.2227	0.2404	0.5457	0.2506	-0.4545	0.2455
L.insurance.insurance	-0.6212	0.2896	1.1246	0.3179	1.1571	0.2924
L.insurance.retirement	-0.5281	0.2418	0.8338	0.2635	0.5474	0.2855
L.retirement.retirement	-0.3881	0.3167	-0.077	0.2613	0.0021	0.4468
SD.JobASC	9.0912	1.4289	7.5067	1.1349	8.9915	1.4031
SD.wage	0.2943	0.0466	0.3718	0.0511	0.3189	0.0544
SD.layoff	0.0272	0.0101	0.0358	0.0132	0.0314	0.0123
SD.exposure	0.0832	0.0146	0.0894	0.0163	0.0866	0.0156
SD.insurance	0.9257	0.2654	1.3423	0.3186	1.2328	0.3065
SD.retirement	1.1959	0.2685	1.3804	0.2971	1.1905	0.275
